# Isolation and Characterization of a *Psathyrostachys huashanica* Keng 6Ns Chromosome Addition in Common Wheat

**DOI:** 10.1371/journal.pone.0053921

**Published:** 2013-01-10

**Authors:** Wanli Du, Jing Wang, Yuhui Pang, Yanli Li, Xinhong Chen, Jixin Zhao, Qunhui Yang, Jun Wu

**Affiliations:** Shaanxi Key Laboratory of Genetic Engineering for Plant Breeding, College of Agronomy, Northwest A&F University, Yangling, Shaanxi, China; Ben-Gurion University, Israel

## Abstract

The development of alien addition lines is important for transferring useful genes from exotic species into common wheat. A hybrid of common wheat cv. 7182 (2*n* = 6*x* = 42, **AABBDD**) and *Psathyrostachys huashanica* Keng (2*n* = 2*x* = 14, **NsNs**) via embryo culture produced the novel intergeneric disomic addition line 59-11. The seed morphology of 59-11 resembled the parent 7182 and it exhibited extreme agronomic characteristics, i.e., twin stable spikelets, fertile florets, and multi-kernel clusters. Furthermore, 59-11 produced plump kernels with a high seed-setting percentage during the advanced maturation stage. The line was screened based on genomic *in situ* hybridization, EST-SSR, EST-STS, and gliadin to identify *P. huashanica* chromatin in the wheat background. The chromosome number and configuration of 59-11 was 2*n* = 44 = 22 II and we confirmed the 6**Ns** disomic chromosome additions based on A-PAGE analysis and molecular markers. The results suggested that the production of twin spikelets and multiple kernels per spike in the wheat-*P. huashanica* addition line was related to homologous group 6 in the wheat chromosome. This is the first report of the introduction of improved spike traits into common wheat from the alien species *P. huashanica* and it opens up the possibility of increasing the wheat yield based on this enlarged gene pool.

## Introduction

The enrichment of wheat (*Triticum aestivum*) cultivars requires the continuous supply of new genetic variation [1]. The wild relatives of common wheat have been used for this purpose since the last century. Many genes that confer exceptional traits have been introduced into wheat by wide hybridization [2], [3], [4]. A rapid and effective method applied during the transfer process is the production of a tertiary gene pool, such as an addition line. Indeed, the production of alien addition lines is an important step during successful gene transfer to wheat because it plays a bridging role in the creation of alien substitution lines and translocation lines. Useful traits have been introduced successfully from wild relatives, including *Thinopyrum elongatum* [5], *Secale cereale* L. [6], *Leymus racemosus* [7], and *Hordeum chilense* [8], into hexaploid wheat via the development of alien addition lines.

*Psathyrostachys huashanica* Keng (2*n* = 2*x* = 14, **NsNs**) is a perennial cross-pollinating plant (subfamily *Pooideae,* tribe *Triticeae,* family *Poaceae*), which is generally found on the residual soil of mountainous braes and rocky slopes in the Huashan Mountains, Shaanxi Province, China [9], [10], [11]. It is characterized by early maturity and a high resistance to biotic (scab, stripe rust, take-all, and powdery mildew) and abiotic (cold, salinity, drought, and barren conditions) stresses [11], [12], [13], [14], [15], [16], [17]. *P. huashanica* Keng is also an important germplasm resource used for research into the floristic composition, origin, evolution, and breeding of *Triticum* spp.

Our research team successfully produced a heptaploid hybrid H8911 of common wheat and *P. huashanica* via embryo culture and back-crossing [12]. After a second back-crossing and one generation of selfing, we then developed a batch of wheat-*P. huashanica* monosomic addition lines [13]. Extensive studies of *P. huashanica* have also produced intergeneric amphiploids [14], [16], disomic addition lines [17], a 1 **Ns** addition line [15], a 3 **Ns** addition line [18], and disomic and monosomic addition lines [19]. However, there are no reports of 6 **Ns** alien disomic addition lines. The objectives of the current study were: (a) to identify the alien chromosome in wheat lines derived from crosses with *P. huashanica* using EST-SSR, EST-STS, and gliadin analyses; and (b) to describe and map the characteristic ‘twin spikelets’ derived from *P. huashanica*.

## Results

### Spike Traits of the Progeny Line Derived from Wheat-*Psathyrostachys huashanica*

**Figure 1:**
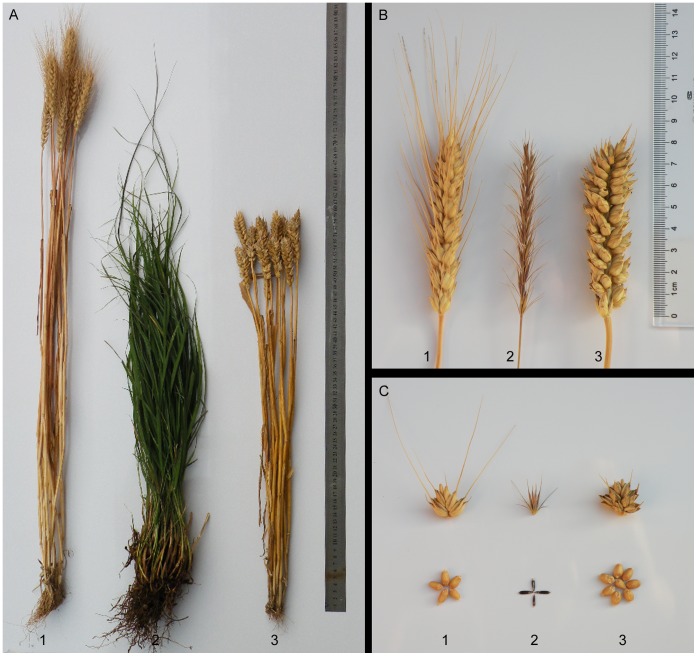
Morphological comparison of adult plants, spikes, spikelets, and seeds from disomic addition line 59-11 and its parents, wheat cv. 7182 and *Psathyrostachys huashanica*. a adult plants; **b** spikes; **c** spikelets and seeds. **1** 7182, **2** *Psathyrostachys huashanica*, **3** 59-11.

The progeny line 59-11 derived from a cross between common wheat cv. 7182 and *P. huashanica* had interesting morphotypes, i.e., a stable genetic character with twin spikelets, and multiple florets and kernels. The twin spikelets emerged in the lower middle position of a spike and the total grain number per twin spikelet was >7. The spikes of the 59-11 plants were equipped with small tip awns and they were located in a similar position to that in *P. huashanica*, which distinguished them from the full awn of the female parent 7182. The 59-11 spikes were bulkier than the spikes of its parents. In addition, 59-11 produced plump red seeds that were similar to those produced by the female parent 7182 and they had a higher 1000-kernel weight (about 48 g) (Fig. 1). The plant height of 59-11 was lower than that of its parents, wheat cv. 7182 and *P. huashanica*. Ten plants were used to determine the mean height of 59-11 and they contained 150 tillers with an average of 15 tillers per plant, while we also analyzed the length, kernels, and spikelet numbers of 150 spikes. The Student’s *t* test showed that the 59-11 was significantly different from its parents, wheat cv. 7182 and *P. huashanica* (*P*<0.01 and *P*<0.05) in terms of the plant height, kernels per spike, and 1000-kernel weight (Table 1).

**Table 1:** Morphological characteristics of 7182, *Psathyrostachys huashanica,* and the 6Ns addition line.

Characters	7182		*P. huashanica*		59-11	
	M	R	M	R	M	R
**Plant height (cm)**	85Aa	(80–91)	80Aa	(75–86)	60Bb	(55–65)
**Tillering**	12Aa	(8–18)	clump	clump	15Aa	(14–18)
**Spike length (cm)**	8Aa	(6–11)	7.5Aa	(5–10)	7.5Aa	(6–10)
**Kernels per spike**	52ABb	(41–60)	40Bb	(21–53)	95Aa	(72–120)
**Spikelet per spike**	16Aa	(12–22)	15Aa	(12–18)	10Aa	(8–12)
**Spikelet per twin spikelets**	–	–	–	–	10	(8–12)
**Kernels per spikelet**	3Aa	(2–5)	3Aa	(2–4)	3Aa	(2–4)
**Kernels per twin spikelets**	–	–	–	–	7	(6–9)
**Thousand-grain weight**	43Ab	(41–45)	3.3Bc	(2.9–4.0)	48Aa	(46–50)
**Awn length (cm)**	4Aa	(1–6)	0.7Aa	(0–1.5)	0.5Aa	(0–1)

M: Mean, R: range, – indicates no data recorded. Significant differences in the means are indicated at the *P*<0.01 (capital letter) and *P*<0.05 (small letter) levels, according to the Student’s *t* test.

### Cytological Characterization of Wheat-*P. huashanica* Disomic Addition Lines

**Figure 2:**
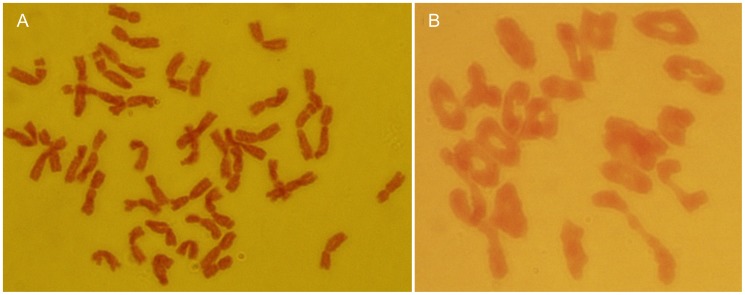
Mitotic and meiotic patterns of the wheat-*Psathyrostachys huashanica* addition line 59-11. **a** Somatic chromosomes in root tips, 2*n* = 44; **b** Chromosome behavior of PMCs at metaphase I, 2*n* = 22 II.

The hybrid H881 (2*n = *28, **ABDNs**) derived from 7182 and *P. huashanica* was successfully produced by embryo culture in 1991 and spontaneous chromosomal doubling via backcrossing, which was conducted to generate the heptaploid hybrid H8911 (2*n* = 49, **AABBDDNs**). Further backcrossing produced a batch of wheat-*P. huashanica* monosomic addition lines. One of these, 59-11, was found to have a chromosome configuration of 2*n* = 43 based on cytogenetic and GISH analyses, while it also possessed the specific A-PAGE band of *P. huashanica* and a twin spikelet morphology. Continuous strict, single-selfing generations were conducted, which were also accompanied by cytology, GISH screening, A-PAGE analyses, and morphological observation. Twenty BC_2_F_2_ plants having a chromosome number of 2*n* = 44 and twin spikelets with multiple florets and kernels were selected (Fig. 2a). We examined the chromosome pairing behavior of these plants in the PMCs during metaphase I. Twenty-two bivalents were found in 93% of the PMCs (Fig. 2b). The progeny line had an average of 0.76 univalents, 18.09 ring bivalents, and 3.15 rod bivalents. No trivalents or quadrivalents were observed (Table 2). These results suggested that the exotic *P. huashanica* chromosome had a high inheritability ratio during the selection of the addition line. Thus, *P. huashanica* chromosomes can be transmitted stably in a wheat background. Finally, we developed the wheat-*P. huashanica* disomic addition line 59-11 with twin spikelets, and multiple florets and kernels.

**Table 2:** Chromosome pairing at metaphase I in PMCs from the disomic addition line 59-11 and its parents, wheat cv. 7182 and *Psathyrostachys huashanica.*

			Chromosome configuration				
			**Univalent**	**Bivalent**			
Materials	2*n*	No. of cells		**Rod**	**Ring**	**Total**	Chiasmata/cell
7182	42	100	0.37	2.84	17.51	20.35	37.90
			(0–3)	(0–4)	(14–21)	(19–21)	(38–42)
*P. huashanica*	14	100	0.08	1.78	12.22	14	24.58
			(0–1)	(0–4)	(9–14)	(13–14)	(12–14)
59-11	44	260	0.76	3.15	18.09	21.24	40.86
			(0–4)	(1–7)	(15–21)	(20–22)	(40–44)

### GISH Analysis of the Wheat-*P. huashanica* Progeny Line

**Figure 3:**
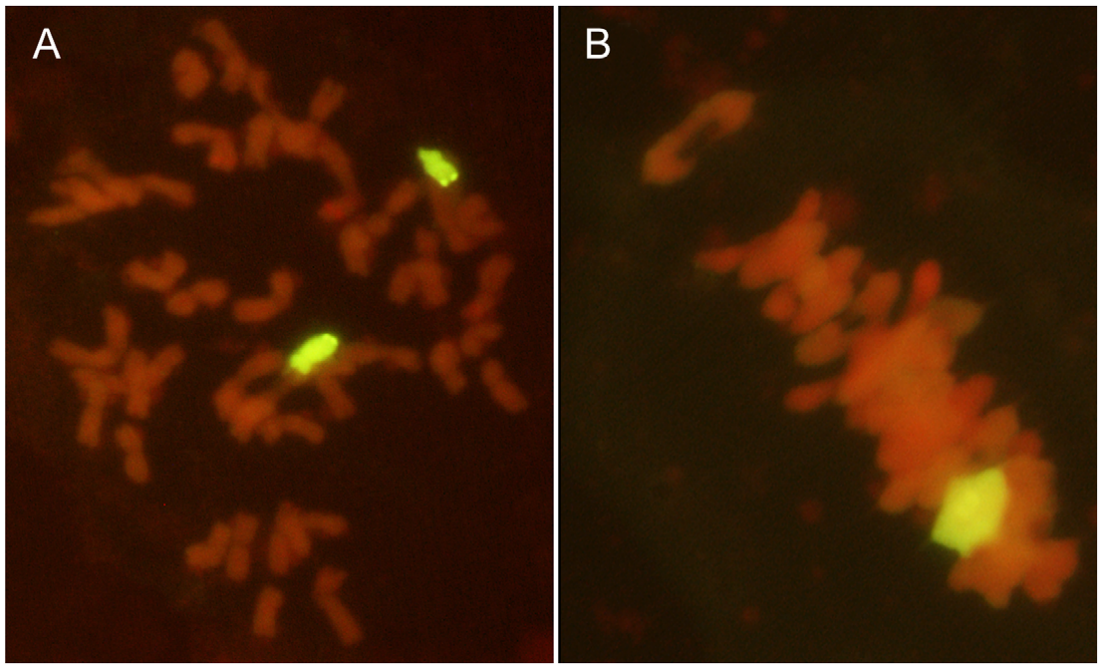
Mitotic and meiotic GISH analysis of the wheat-*Psathyrostachys huashanica* addition line 59-11. **a** Somatic metaphase of the disomic chromosome addition line with two *Psathyrostachys huashanica* chromosomes (yellowish-green color); **b** PMCs at meiotic metaphase I, where the yellowish-green color indicates a ring bivalent chromosome from *Psathyrostachys huashanica* (color figure online).

GISH was used to analyze the chromosome configuration and composition of 59-11. The total genomic *P. huashanica* DNA was used as a probe with Chinese Spring as the blocking DNA. Mitotic GISH showed that the root tip cells of 59-11 contained two chromosomes with strong yellowish-green hybridization signals (Fig. 3a). Meiotic GISH indicated that the metaphase I PMCs also had one ring bivalent yellowish-green signal (Fig. 3b). This suggested that 59-11 contained two chromosomes from different genomes in a wheat background. The two alien chromosomes could form bivalents and they were processed normally during synapsis, pairing, and segregation. These results also confirmed that 59-11 was a *P. huashanica* disomic addition line, which contained 42 wheat chromosomes and a pair of *P. huashanica* chromosomes.

### Characterization with EST-SSR and EST-STS Markers

**Figure 4:**
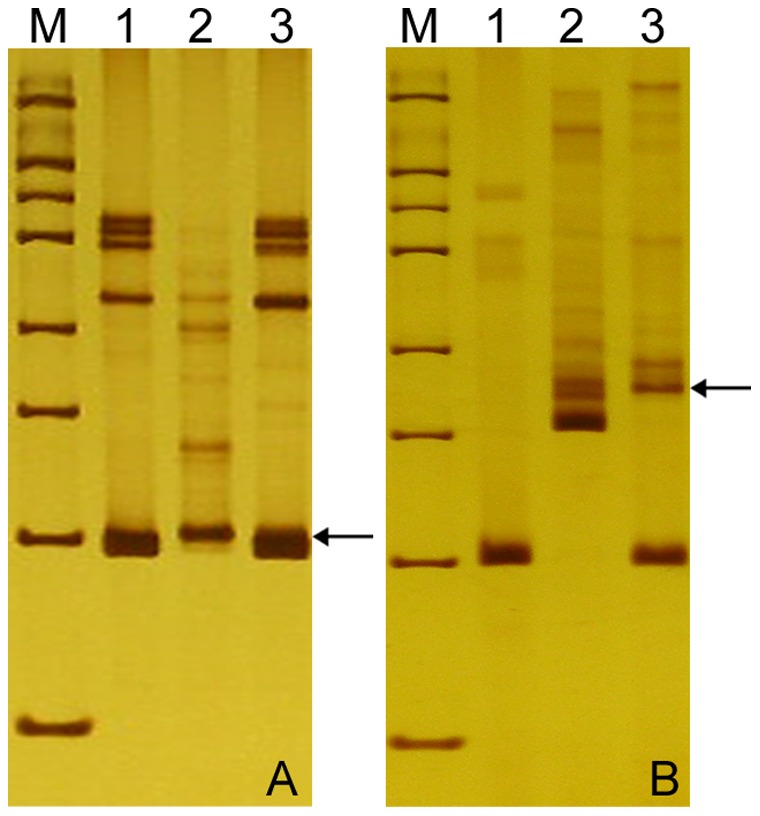
Identification of *Psathyrostachys huashanica* chromosomes using EST-SSR and EST-STS markers in disomic addition line 59-11 and its parents, wheat cv. 7182 and *Psathyrostachys huashanica*. **a** *SWES2* and **b** *CD452568* amplified specific bands in the addition line in chromosomes 6A, 6B, and 6DS, and, 6AL, 6BL, and 6DL, respectively (arrows). **M** marker, **1** *Psathyrostachys huashanica*, **2** 7182, **3** chromosome addition line 59-11. Arrows indicate the diagnostic amplification products of *Psathyrostachys huashanica*.

EST-SSRs and EST-STS markers were used to determine the homoeologous relationships between the added *P. huashanica* chromosomes and wheat. We screened 46 EST-SSRs primer pairs in the parents, which were distributed on different chromosome groups in wheat. We found that 40 pairs (87%) had amplified polymorphism bands. However, when these primers were used to analyze the disomic wheat-*P. huashanica* line 59-11, only one EST-SSR, *SWES2*, which was located on 6**A**, 6**B**, and 6**DS**, produced the specific bands in *P. huashanica* and 59-11 (Fig. 4a; Table 3). This suggests that the pair of alien *P. huashanica* chromosomes transferred into common wheat were associated with the sixth group of wheat chromosomes.

**Table 3:** Primers used to produce specific markers for chromosome 6Ns in *Psathyrostachys huashanica.*

Marker	Type	EST accession No.^b^	Primer	Product size (bp)	Location	Annealing temperature (°C)
*Swes2* a	EST-SSR	Chen et al. (2005)	F: TGGACCCCGAGCATAACA	250/255/244	6A 6B 6DS	60
			R: CCCCAACACCGCAATCTA			
CD452568	EST-STS	CD452568	F: TTTGCATTTTCGTCTGCAAG	429	6AL 6BL 6DL	60
			R: TCGACACGAGCAAGATTCAC			

a The marker *Swes2* was previously mapped by Chen et al. (2005) and it was verified in this study.

b The EST Accession No. is from the following database: http://wheat.pw.usda.gov/SNP/new/pcr_primers.shtml.

We used 200 EST-STS primer pairs related to seven groups of wheat chromosomes to gain further insights into 59-11. One EST-STS primer, *CD452568*, that mapped onto 6**AL**, 6**BL**, and 6**DL**, amplified the allele-specific bands of *P. huashanica* in the disomic addition line 59-11(Fig. 4b; Table 3). These results confirmed that the two *P. huashanica* chromosomes in addition line 59-11 were homoeologous to the sixth chromosome group in wheat.

### Gliadin Analysis

**Figure 5:**
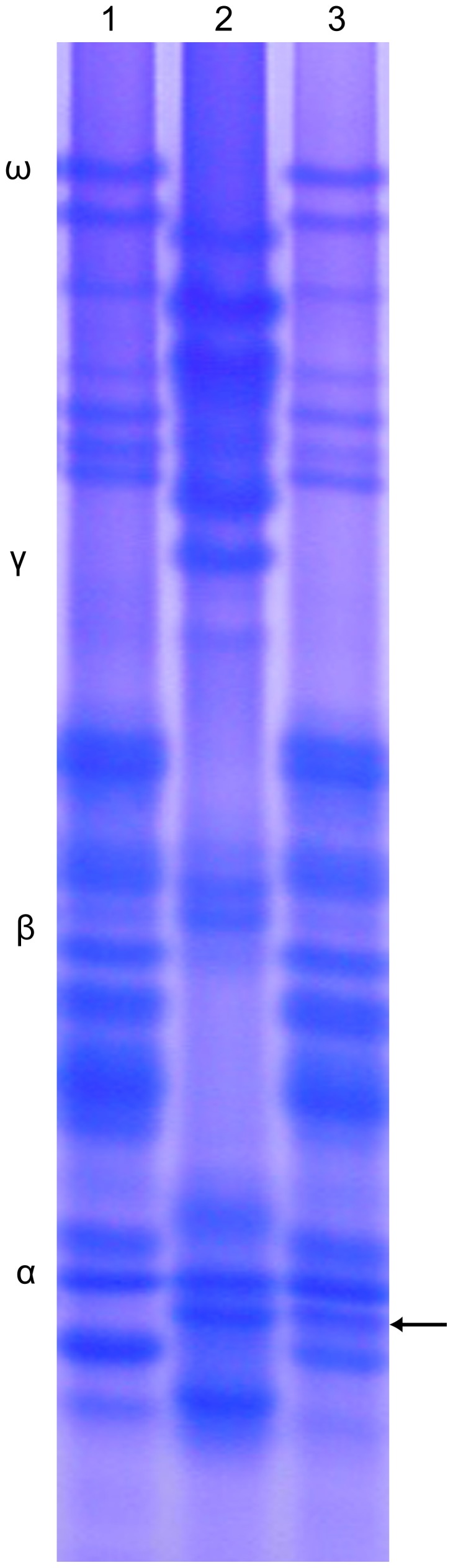
A-PAGE analysis of the disomic addition line and its parents, wheat cv. 7182 and *Psathyrostachys huashanica*. **1** 7182, **2** *Psathyrostachys huashanica*, **3** 59-11. The arrow shows specific bands from *Psathyrostachys huashanica* that appeared as α-gliadin in 59-11.

A-PAGE was used to separate gliadin proteins from the *P. huashanica* and chromosome disomic addition line 59-11. One band that was specific to *P. huashanica* in the α-gliadin section, based on its mobility, was also detected in 59-11 (Fig. 5). It was clear that 59-11 had the same banding patterns as common wheat 7182, while it also contained a specific α-gliadin band from *P. huashanica*. Given that the encoded α-gliadin genes mapped onto the short wheat chromosome arm of group 6, the specific *P. huashanica* α-gliadin band further confirmed that the *P. huashanica* chromosome in 59-11 was correlated with the sixth group of the wheat chromosome.

## Discussion

### Identification of *P. huashanica* Chromosomes in the Addition Line

GISH is the most efficient and accurate technique for tracking alien chromosomes or smaller fragments of different genomes in allopolyploids and wide hybrids [20]. GISH was employed to identify the chromosomal configuration and composition of the 59-11 line with exceptional spikelet characteristics during the mitotic and meiotic phases, using *P. huashanica* as a probe and Chinese Spring as a blocker. The results showed that the progeny line was a wheat-*P. huashanica* disomic addition line (Fig. 3). We tested the segregation of the *P. huashanica* chromosome in the progeny line 59-11 and we found that the desirable spike characteristics had a close relationship a pair of *P. huashanica* chromosomes. The wheat-*P. huashanica* addition line from 59-11 was also shown to be homologous to the group 6 wheat chromosome based on EST-SSR, EST-STS, and A-PAGE analysis (Fig. 4; Fig. 5). It was clear that the chromosomes derived from *P. huashanica* were the main cause of the increased spikelet number and multiple kernels per spike, and the chromosomes were assigned as 6**Ns**. This is the first report of a useful and effective agronomic trait involved with enhanced floret and kernel number being produced in a common wheat background via the transfer of chromatins from the alien species *P. huashanica*. This wheat-*P. huashanica* disomic addition line with exceptional agronomic traits could provide fresh germplasm for wheat yield improvement.

The EST regions, including intron sequences, could also be used as accurate anchor markers of genomic relationships when comparing the homoeologous chromosome regions [21]. Furthermore, EST-SSR and EST-STS markers have been used extensively as effective tools for the genetic analysis of alien chromosomes. The alien chromatin of wheat-*A. cristatum* addition and substitution lines were designated as 6**P** based on EST-SSR analysis [22]. An alien fragment on 2**DL** in a wheat-*Thinopyrum* substitution line was detected by EST-STS [23]. Two sets of EST-SSR markers were used to identify the sub-arm of prolamin loci on the 1**H^ch^** chromosome in a wheat background [8]. One EST-SSR marker and one EST-STS marker in wheat homoeologous group 6 identified polymorphism differences between wheat and *P. huashanica*, suggesting that these loci were physically located in similar homoeologous positions in *P. huashanica* and its progeny line. In addition, A-PAGE was used to determine the homoeologous group and genome affinity of the added chromosomes.

Many novel storage protein genes have been found in genera that are genetically close to wheat, e.g., *P. huashanica* [14], [15], *Th. intermedium* ssp. *trichophorum* [24], and *C. delieana* [25]. We found that the disomic addition line 59-11 had a specific band from *P. huashanica* in the α-gliadin section based on gliadin analysis (Fig. 5). The α- and β-gliadins are encoded by the short arm of group 6 in wheat, so it was inferred that the 59-11 alien disomic addition line carried the group 6 homoeology chromosome 6**Ns** from *P. huashanica*. Thus, the gliadin band of *P. huashanica* can be used as genetic marker to detect the corresponding chromosomes regions during gene transfers to wheat. It was concluded that the alien disomic addition line carried the group 6 homoeology chromosome 6**Ns** from *P. huashanica* based on a combination of EST-SSR, EST-STS, and A-PAGE analysis. These primers and bands could be used as simple and convenient markers for tracing *P. huashanica* 6**Ns** in a wheat background.

### Potential Value of Twin Spikelets with Greater Numbers of Florets and Grains

The wheat yield could be improved by increasing the wheat grain number per unit area and per spike, and there is a growing interest in increasing the spikelet number per spike [26]. This superior spike trait could be useful for improving the yield of wheat and it can be achieved by the introgression of chromosomes from wild relatives of wheat, e.g., *Secale cereale* L. [27], [28] and *Hordeum chilense* [29]. In the current study, the addition line 59-11 was identified as a 6**Ns** chromosome addition line, which produced twin spikelets and an average of ten twin spikelets per spike. 59-11 was characterized by fertile florets, multiple kernels, a more advanced mature stage, plump kernels, and an optimum combination of tiller number and high seed-setting percentage, which possibly makes it optimal for high-yield breeding (Table 1, Fig. 1).

In summary, we found that gene(s) controlling the twin spikelets per spike characteristic of the wheat-*P. huashanica* progeny line were located on the 6**Ns** chromosome. Chromosome 6**D** was active in improving the floret number per spike by increasing the spikelet number within a spike [30]. Wheat-*A. cristatum* chromosome addition lines and substitution lines related to 6**P** also have more florets and kernels within a spike [22]. *P. huashanica*, as a tertiary gene pool, has homologous genomes with common wheat [31]. It is possible that the exceptional characteristics of the spike could be enhanced further by doubling the genes or accumulating genes in the group 6 loci. Ongoing research will be needed to monitor the association between the different arms of 6**Ns** and the exceptional spike trait to locate and/or map this trait onto specific chromosome regions. This novel addition line provides a solid foundation for the introgression of critical genes/QTLs from 6**Ns** into wheat.

A combination of GISH, EST-SSR, EST-STS, gliadin, and morphological analyses clearly identified the presence of the *P. huashanica* chromosome 6**Ns** in a wheat background, where it was responsible for the production of twin spikelets per spike in wheat. The results of this study may be important for further elucidating the structure and function of the *P. huashanica* 6**Ns** chromosome in a wheat background, while it also provides fresh insights into the evolutionary divergence of *P. huashanica* chromosomes from its cereal relatives.

## Materials and Methods

### Plant Materials

Wheat cv. 7182 (2*n = *42, **AABBDD**), *P. huashanica* (2*n = *14, **NsNs**), and the progeny of 59-11 (2*n = *44) were used in this study. The parents 7182 and *P. huashanica* were included as controls to assess the spike traits and for EST-SSR, EST-STS, and gliadin analysis. Chinese Spring was used as a source of blocking DNA in the genomic *in situ* hybridization (GISH) analysis. These plant materials are deposited at the Shaanxi Key Laboratory of Genetic Engineering for Plant Breeding, College of Agronomy, Northwest A&F University, Shaanxi, China.

### Evaluation of Spike Traits

The association of *P. huashanica* chromosomes with twin spikelets was evaluated during the 2009, 2010, and 2011 field harvest seasons in Yangling, Shaanxi, China (N 34°16′ 56.24′′, E 108°4′ 27.95′′). This area has fertile soil and a temperate continental monsoon climate, with an elevation of 530.1–403.2 m, and the annual average temperature, sunshine, and precipitation are 12.9°C, 2196 h, and 660 mm, respectively. Plants were arranged separately in a randomized complete block design using two replicates. Ten randomly selected 59-11 plants to assess the yield-related traits such as tillering, spike length, kernel number per spike, the number of spikelets, number of seeds per spikelet, the 1,000-kernel weight, awn length and plant height. The Student’s *t* test was used to test for significant differences between 59-11 and its parents, wheat cv. 7182 and *P. huashanica,* for all traits, except for the spikelets per twin spikelet and the kernels per twin spikelet.

### Cytogenetic Analysis

Seeds were germinated in Petri dishes at 23°C in the dark until the roots reached 1–2 cm in length. Roots were excised from geminated seeds and incubated in ice-cold water overnight, and fixed in Carnoy’s solution with 95% ethanol-acetic acid (3∶1, v/v). Pollen mother cells (PMCs) were collected from young panicles and fixed in absolute ethanol-chloroform-glacial acetic acid (6∶3:1, v/v). Mitotic and meiotic chromosomes were squashed on a slide in a drop of acetocarmine and 45% acetic acid, and then used for cytological observation and GISH, respectively. The cover slips were removed after freezing the slides with liquid nitrogen, followed by air-drying and storage at –20°C.

### GISH Analysis

The total genomic DNA was extracted from the fresh leaves of *P. huashanica* using the modified CTAB method [32]. The *P. huashanica* probe DNA was labeled with digoxigenin (digoxigenin-11-dUTP, Roche, Germany) via the nick-translation method. *In situ* hybridization was performed as previously described [33]. The slides were treated with RNase A (2 µg/ml in ddH_2_O) for 1 h at 37°C. The 40 µl hybridization solution contained 1 µl 10% (W/V) SDS, 1 µl ssDNA (salmon sperm DNA, 5 ug/µl), 20 µl formamide, 4 µl 20× SSC, 8 µl 50% (W/V) dextran sulfate, 100 ng probe DNA, and 3 µl ddH_2_O, and it was denatured by boiling for 10 min, before cooling in ice for 10 min. The denatured hybridization mixture was applied to each slide and allowed to hybridize overnight in a humid chamber at 37°C. Two post-hybridization washes of 2× SSC at 37°C were applied for 3 min each, followed by 3 min washes at room temperature. We added 50 µl FITC coupled to anti-dig antibody to detect and visualize the labeled chromosomes, while propidium iodide (PI) was added to stain unlabeled chromosomes. Fluorescent signals were viewed and photographed (Olympus BX60) with a Photometrics SenSys CCD camera.

### EST-SSR and EST-STS Molecular Analysis

Genomic DNA was isolated from the wheat-*P. huashanica* addition line and both parents, as previously described [32]. To characterize the genomic composition of the wheat-*P. huashanica* addition line, we used 46 EST-SSR and 200 EST-STS multiple-loci primer pairs that were derived from a previous report [34] and (http://wheat.pw.usda.gov/SNP/new/pcr_primers.shtml), respectively, which were located on seven wheat homoeologous groups. Twenty microliters of each reaction mixture contained 2 µl 10× PCR buffer, 2 µl primer (2.5 µmol/ml), 2 µl DNA template (40–60 ng/µl), 1.6 µl dNTPs (2.5 µmol/ml), 1.6 µl MgCl_2_ (2.5 mmol/ml), 0.1 µl *Taq* polymerase (5 U/µl), and 10.7 µl ddH_2_O. The amplification procedure consisted of initial denaturation for 3 min at 94°C, followed by 35 cycles of 1 min at 94°C, 50 s at 60°C, and 1 min at 72°C, with a final extension for 10 min at 72°C. The PCR products were separated under standard conditions on 8% non-denatured polyacrylamide gel electrophoresis (PAGE) gels and visualized by silver staining.

### Prolamin Extraction and Electrophoretic Analysis

Seed proteins were extracted from the crushed endosperms of single kernels. Acid polyacrylamide gel electrophoresis (A-PAGE) was conducted to fractionate gliadin proteins using a previously described method [15]. The extraction buffer contained 25% (v/v) 2-chloroethanol and 0.05% (w/v) methyl green, and the separations were conducted using standard A-PAGE at pH 3.1 with low catalyst (ferrous sulfate and hydrogen peroxide) levels to increase the gel firmness. Electrophoresis was carried out at 20 mA/gel at 10°C. The gels were fixed with 15% (w/v) trichloroacetic acid solution for 30 min, then stained overnight with 0.1% (w/v) Coomassie Brilliant Blue R-250 dissolved in absolute ethanol. De-staining was performed with tap water.
